# Analysis of super-enhancer using machine learning and its application to medical biology

**DOI:** 10.1093/bib/bbad107

**Published:** 2023-03-23

**Authors:** Ryuji Hamamoto, Ken Takasawa, Norio Shinkai, Hidenori Machino, Nobuji Kouno, Ken Asada, Masaaki Komatsu, Syuzo Kaneko

**Affiliations:** Division Chief in the Division of Medical AI Research and Development, National Cancer Center Research Institute; a Professor in the Department of NCC Cancer Science, Graduate School of Medical and Dental Sciences, Tokyo Medical and Dental University and a Team Leader of the Cancer Translational Research Team, RIKEN Center for Advanced Intelligence Project; Cancer Translational Research Team, RIKEN Center for Advanced Intelligence Project and an External Research Staff in the Medical AI Research and Development, National Cancer Center Research Institute; Department of NCC Cancer Science, Graduate School of Medical and Dental Sciences, Tokyo Medical and Dental University; Cancer Translational Research Team, RIKEN Center for Advanced Intelligence Project and an External Research Staff in the Medical AI Research and Development, National Cancer Center Research Institute; Department of Surgery, Graduate School of Medicine, Kyoto University; Cancer Translational Research Team, RIKEN Center for Advanced Intelligence Project and an External Research Staff of Medical AI Research and Development, National Cancer Center Research Institute; Cancer Translational Research Team, RIKEN Center for Advanced Intelligence Project and an External Research Staff of Medical AI Research and Development, National Cancer Center Research Institute; Division of Medical AI Research and Development, National Cancer Center Research Institute and a Visiting Scientist in the Cancer Translational Research Team, RIKEN Center for Advanced Intelligence Project

**Keywords:** machine learning, super-enhancer, ChIP-seq, high-order chromatin structure, precision medicine

## Abstract

The analysis of super-enhancers (SEs) has recently attracted attention in elucidating the molecular mechanisms of cancer and other diseases. SEs are genomic structures that strongly induce gene expression and have been reported to contribute to the overexpression of oncogenes. Because the analysis of SEs and integrated analysis with other data are performed using large amounts of genome-wide data, artificial intelligence technology, with machine learning at its core, has recently begun to be utilized. In promoting precision medicine, it is important to consider information from SEs in addition to genomic data; therefore, machine learning technology is expected to be introduced appropriately in terms of building a robust analysis platform with a high generalization performance. In this review, we explain the history and principles of SE, and the results of SE analysis using state-of-the-art machine learning and integrated analysis with other data are presented to provide a comprehensive understanding of the current status of SE analysis in the field of medical biology. Additionally, we compared the accuracy between existing machine learning methods on the benchmark dataset and attempted to explore the kind of data preprocessing and integration work needed to make the existing algorithms work on the benchmark dataset. Furthermore, we discuss the issues and future directions of current SE analysis.

## INTRODUCTION

On 14 April 2003, almost 50 years after Watson and Crick published the deoxyribonucleic acid (DNA) double-helix structure, the complete human genome sequence, analyzed in the Human Genome Project, an international consortium, was published [1–3]. Ever since this announcement, the world has entered the post-genome era, and expectations for genomic medicine and the application of genome information to medical treatment have increased; various studies have been actively conducted to meet this goal. In particular, when the ‘Precision Medicine Initiative’ was announced by the US President Barack Obama in his State of the Union address at the beginning of 2015, the promotion of precision medicine was determined to be one of the most important healthcare policies worldwide, and a large budget was allocated to this goal [4–6]. Consequently, Memorial Sloan Kettering-Integrated Mutation Profiling of Actionable Cancer Targets (MSK-IMPACT) and FoundationOne CDx, tumor profiling tests for solid tumors based on next-generation sequencing (NGS)-based genetic mutation analysis, were approved by the US Food and Drug Administration (FDA) in 2017, thereby promoting the application of optimal medicine based on genetic information not only at the research level but also in clinical practice [7, 8]. The clinical application of precision medicine is generally based on tumor profiling tests to provide optimal anticancer drugs; however, the number of patients who can benefit from precision medicine is limited, which is problematic [9–11]. For example, in lung adenocarcinoma, mutations in several genes, such as EGFR, play a major role in tumorigenesis (called driver mutations), and the development of molecularly targeted drugs against these mutations is in progress [12–14]. On the contrary, a certain number of patients with lung adenocarcinoma, referred to as ‘pan-negative’ cases with no detectable driver mutations, also exist, making it difficult to provide appropriate therapeutic agents [15, 16]. The elucidation of the true nature of cancer is an unmet need for developing treatment strategies for these driver-negative cancer cases.

Under these circumstances, the role of super-enhancers (SEs) in the oncogenic process has garnered attention. SEs are genomic structures that strongly induce gene expression and reportedly establish gene expression networks that underlie cell malignancy signaling [17]. For instance, TP63 is a master transcription factor that leads to the onset of squamous cell carcinoma, and, together with coactivators, such as bromodomain-containing protein 4 (BRD4), forms a SE consisting of giant molecular assemblies, and TP63 itself is reported to be induced by the SE [18, 19]. The identification of SEs is important because aberrant gene expression due to SE formation is difficult to be identified by conventional genomic analysis techniques for detecting mutations in genomic coding regions and thus has the potential to broaden the patient population that would benefit from precision medicine. Technically, the identification of SEs requires mainly chromatin immunoprecipitation-sequencing (ChIP-seq) analysis, integrated analysis with transcriptome data; from the perspective of the scrutiny of the mechanism, an integrated analysis with high-throughput chromosome conformation capture (Hi-C) analysis and whole-genome analysis has been necessary. This generally requires advanced techniques and complicated analysis steps. In addition, because of the high cost of analytical methods, analysis using a large number of clinical specimens requires a large budget, and a series of analyses can be performed only in a limited number of laboratories. To stimulate a wide range of SE research in the future and to aim for a clinical application of the obtained results, it is important to construct an analysis platform with high robustness whose performance can be generalized.

In this regard, researchers have utilized machine learning techniques at the core of artificial intelligence (AI) in analyzing SEs and integrating them with other data. Currently, machine learning is being utilized in the field of medical biology, and several important results have been published: findings on the analysis of medical images, such as endoscopic, radiological and pathological images; the findings of omics analysis; and the findings of the analysis of medical information using natural language processing [20–32]. The results are not merely presented at the research level; the US FDA has approved many AI-based medical devices, and the number of AI-based medical devices that have been clinically applied continues to grow [33–38]. Machine learning is being used not only for genome analysis but also for epigenetic analysis and chromatin structure analysis [39–42]; machine learning is speculated to have been integrated for SE-related analysis.

Therefore, in this review, we introduce the history of SEs and their methods of analysis, and the state-of-the-art SE analysis, utilizing machine learning and the results of integrating analysis with other data. In addition, we compared the accuracy between existing methods on the benchmark dataset and attempted to explore what kind of data preprocessing and integration work would be required to make the existing methods work on the benchmark dataset. Furthermore, by discussing current issues and future directions, we aim to provide a comprehensive understanding of the current status of SE analysis in medical biology.

### History of SEs

**Figure 1:**
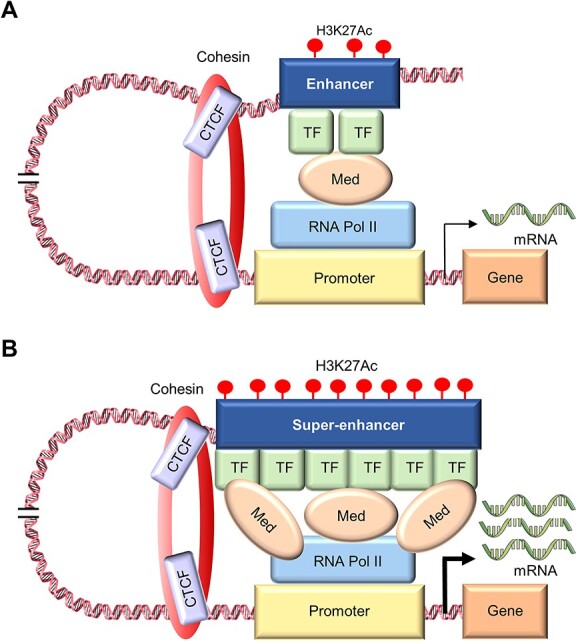
Conceptual diagram of enhancer (**A**) and SE (**B**). Red circles indicate histone mark H3K27Ac. Abbreviations: TF, transcription factor; Med, Med1 (Mediator Complex Subunit 1); RNA pol II, RNA polymerase II.

The regulation of transcription by enhancers has been studied since the 1980s [43]. Enhancers in genetics are considered short (50–1500 base pairs) DNA regions to which proteins (activators) bind to increase the likelihood of transcription of a particular gene [44, 45]. Often, the proteins that bind to these enhancers are called transcription factors. Enhancers act in *cis* [46] and can be located up to a million base pairs away from the gene or upstream of the transcription start site [47–49]. There are hundreds of thousands of enhancers in the human genome [50, 51]. In further analysis of transcriptional regulation mechanisms by enhancers, large or multicomponent transcriptional regulators with various mechanistic properties were observed, including locus control regions, clustered orifice control elements and transcription initiation platforms (Figure 1A).

In 2013, Richard A. Young’s group found a large and potent enhancer region in the expression regulatory region of genes that establish embryonic stem cell identity, such as *Oct4*, *Sox2* and *Nanog* (Figure 1B), and named the region an ‘SE,’ which led to the use of the term ‘SE’ in the academic field [52]. SEs are a class of regulatory regions that are unusually enriched for binding of transcriptional activators, particularly Mediator Complex Subunit 1 (Med1), and, in mouse embryonic stem cells (mESCs), SEs are defined by Young *et al*. as follows [52]:

(1) By ChIP-seq, the sites where all three master regulators, Oct4, Sox2 and Nanog, bound were considered enhancers. (2) Connect enhancers within 12.5 kb of each other to define a single enhancer that spans a genomic region. (3) The linked enhancers and the remaining individual enhancers (those without adjacent enhancers within 12.5 kb) were ranked based on the background normalization level of the Med1 signal within the genomic region. A small fraction (<3%) of these enhancer regions with Med1 levels above the cutoff were designated as SEs. The remaining enhancer regions were considered ‘typical’ enhancers (TEs).

SEs span large genomic regions, and their median values are generally of an order of magnitude larger than that of TEs (8667 bp versus 703 bp for mESCs) [17, 52–54]. In addition, the SE region generally contains many factors associated with enhancer activity, including ribonucleic acid (RNA) polymerase II (RNA Pol II), RNA from transcribed enhancer loci (eRNA), histone acetyltransferases p300 and CBP, chromatin factors, such as cohesin, histone modifications histone H3 lysine 27 acetylation (H3K27Ac), H3 lysine 4 di-methylation (H3K4me2), H3 lysine 4 mono-methylation (H3K4me1), increased chromatin accessibility measured by DNase-seq and several factors associated with enhancer activity have been shown to be more enriched than in TEs [53].

Not only are SEs biologically important but also abnormalities in their function are reportedly associated with cancer, type I diabetes and Alzheimer’s disease [17]. Especially in cancer, SEs may be potentially important in the misregulation of gene expression. During tumorigenesis, malignant cells acquire SEs in key oncogenes, and higher levels of transcription of these genes than that in healthy cells have also been reported [17, 54–57]. For example, SEs are quite CDK7-dependent, and, in cancer, the treatment of cells with the CDK7 inhibitor THZ1 reduces the recruitment of SE-driven oncogenic transcription factors and suppresses the expression of these associated oncogenes [58–61]. Genome-wide analysis has also shown that BRD4, a reported therapeutic target in cancer, binds preferentially to the SE [62, 63]. Interestingly, the treatment of multiple myeloma cells with the anticancer BRD4 inhibitor JQ1 resulted in the preferential loss of BRD4 at the SE, prompting transcriptional elongation defects that preferentially affect genes with SEs, such as MYC oncogene [54]. Considering that SEs are important oncogenic drivers in many other tumor cells, strategies that target the components of SEs in a variety of tumors may lead to effective cancer therapy [64–70].

Moreover, several online databases that provide datasets on SEs and are intended to support and promote research in this area are also available and can be utilized by researchers working on SEs (Table 1).

**Table 1:** List of databases with information on SEs

No.	Database name	Description	URL	References
1	SEdb 2.0	SEdb 2.0 is a comprehensive SE database that aims to provide many resources on human and mouse SEs. The database is annotated for potential functions of SEs in TF gene regulation. A total of 2670 samples, including 1739 human and 931 mouse samples, with a total of 1,717,744 SEs have been recorded in SEdb 2.0 (as of October 2022). In addition, SEdb 2.0 provides detailed genetic and epigenetic annotation information on SEs; they include information on common SNPs, motif variations, expression quantitative trait loci (eQTLs), risk SNPs, transcription factor-binding sites (TFBSs), CRISPR/Cas9 target sites, DNase I hypersensitivity sites (DHSs), chromatin accessibility regions, methylation sites and chromatin interaction regions and TADs. In summary, SEdb 2.0 can help to elucidate the functions associated with the SE and to identify potential biological effects.	http://www.licpathway.net/sedb/	[145]
2	SEA version 3.0	SEA version 3.0 is a comprehensive archive on SEs, a comprehensive web-based resource dedicated to the collection, storage and online analysis of SEs. SEA version 3.0 also provides an information dataset on SEs to support and promote research in this area. In particular, it provides a scalable and flexible landscape for displaying SE information on a genome scale. Currently, SEA version 3.0 consists of SEs for 11 organisms (as of October 2022); these include 104,428 human SEs, 23,969 mouse SEs, 497 *Drosophila melanogaster* SEs, 131 *Caenorhabditis elegans* SEs, 2922 zebrafish SEs, 5883 chicken SEs, 5794 chimp SEs, 13,442 rhesus SEs, 887 sheep SEs, 284 *Xenopus tropicalis* SEs and 1289 stickleback SEs. SEA version 3.0 allows these datasets to be analyzed, charted, downloaded and easily visualized in the genome browser (SEA-Browser).	http://sea.edbc.org/	[146–148]
3	dbSUPER	dbSUPER, organized by China National Center for Bioinformation (CNCB)-National Genomics Data Center (NGDC), is the first integrated and interactive database of 82,234 SEs in 102 human and 25 mouse tissues/cells. Data can be easily sent to Galaxy instances and GREAT and Cistrome web servers for downstream analysis, visualization in the UCSC Genome Browser and automatic addition of custom tracks. dbSUPER also lists genes associated with SEs and links to external databases, such as GeneCards, UniProt and Entrez. In addition, dbSUPER includes an overlap analysis tool to annotate user-defined regions.	https://asntech.org/dbsuper/	[149]

### Methods for analyzing SEs

Research by Richard A. Young’s group in 2013 provided specific methods for the analysis of SEs [52, 54]. To identify SEs, Young’s group first ranked all enhancers in a cell type by increasing the total background-subtracted ChIP-seq occupancy of Med1 and plotted the total background-subtracted ChIP-seq occupancy of Med1 in the units of total million reads per base pair (rpm/bp). When ChIP-seq data on Med1 were not available, the total background-subtracted ChIP-seq occupancy of the master regulator was used instead. These plots reveal a clear point in the distribution of enhancers where the occupancy signal begins to increase rapidly. To define this point geometrically, the data were first scaled so that the *x*- and *y*-axes were in the 0–1 range. The point on the *x*-axis where the line with slope 1 meets the curve is then found, and enhancers above and below this point are defined as SEs and TEs, respectively. Since then, many papers on SE analysis have been published, and although ChIP-seq data targeting master transcription factors and cofactors, such as Med1 and BRD4, are available, the most frequently used ChIP-seq data are those targeting histone H3K27Ac. Rank Ordering of SE (ROSE), a program developed by Young *et al*., is often used to identify SEs from ChIP-Seq data (Table 2) [71–77].

**Table 2:** List of conventional methods to detect SEs

No.	Model name	Description	URL	Reference
1	ROSE	All enhancers in the cell type were ranked by increasing total background-subtractive ChIP-seq occupancy of Med1 and total background-subtractive ChIP-seq occupancy of Med1 in the units of total rpm/bp (reads per million per base pair) was plotted. When Med1 ChIP-seq data were not available, the total background-subtracted ChIP-seq occupancy of the master regulator was used instead. To geometrically define this point, the data were first scaled so that the *x*- and *y*-axes were 0–1. Then, the point on the *x*-axis where the line with slope 1 is tangent to the curve is found.	http://younglab.wi.mit.edu/super_enhancer_code.html	[52, 54]
2	HOMER	The first step is to detect peaks as in any other ChIP-Seq dataset. The peaks found within a given distance are then ‘stitched’ to a larger region (set to 12.5 kb by default). The SE signal for each of these regions is determined by the total number of normalized reads minus the number of input normalized reads. These regions are sorted by the score, normalized by the highest score and the number of estimated enhancer regions, and regions past the point where the slope is greater than 1 are identified as SEs.	http://homer.ucsd.edu/homer/ngs/peaks.html#Finding_Super_Enhancers	[150]

SEs have also been identified using the HOMER bioinformatics analysis suite (Table 2) [78–80]. According to the HOMER-based approach, SEs are defined based on the elevated local sequence read density of such genomic regions, where the individual ChIP-seq peaks of key transcription factors are closer to each other than 12.5 kb [78]. Each ChIP-seq peak or group of peaks is given a read density score, the sorting based on the score value, and those located on a region of graph slope > 1 are defined as SEs [78].

### Analysis of SEs and integrated analysis of SEs with other data using machine learning

#### Analysis of SEs using conventional machine learning techniques

**Figure 2:**
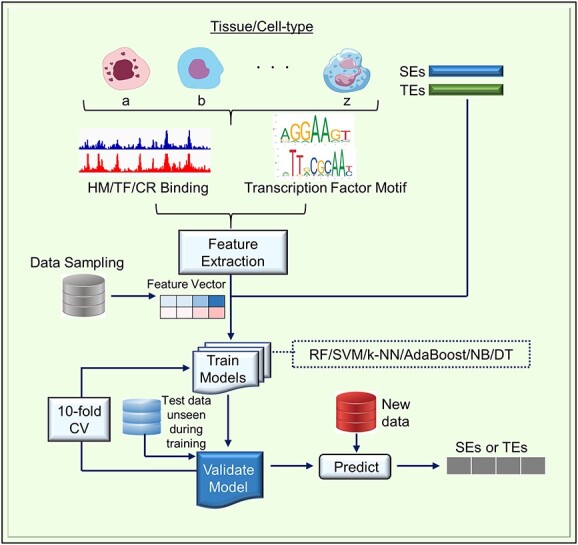
Workflow diagram for Integrated methods for the prediction of SEs (imPROSE) based on Khan and Zhang’s study [81] Abbreviations: HM, Histone Modification; TF, Transcription Factor; CR, Chromatin Regulator; TEs, Typical Enhancers; CV, Cross Validation; RF, Random Forest; SVM, Support Vector Machine; k-NN, k-Nearest Neighbor; AdaBoost, Adaptive Boosting; NB, Naïve Bayes; DT, Decision Tree.

Khan and Zhang integrated diverse publicly available datasets, including histone modifications, ChIP-seq data for chromatin regulators and transcription factors (TFs), DNase I High-sensitivity regions (DHS) and genomic data, to identify key SE signatures and their predictive importance [81]. Importantly, they pioneered the prediction of SEs using six parametric and non-parametric machine learning models: Random Forest (RF), Support Vector Machine (SVM), k-Nearest Neighbor (k-NN), Adaptive Boosting (AdaBoost), Naïve Bayes (NB) and Decision Tree (DT) (Figure 2) [81]. For mESCs, RF had the highest accuracy [area under the curve (AUC) = 0.98] and NB had the lowest accuracy (AUC = 0.85) when predicting SEs using all features of chromatin regulators, TFs, and sequence-specific features. Linear SVM and k-NN showed comparable accuracy (AUC = 0.95), whereas DT achieved an AUC of 0.91. Additionally, no bias was observed in a comparison of nonlinear SVM (Radial Basis Function (RBF) kernel and polynomial kernel) and linear SVM. AdaBoost showed almost the same performance as RF; however, only RF was shown to achieve stable accuracy and repeatability [81]. The models were then compared using individual and different types of features, and each model had feature-type specificity, with linear SVM performing poorly (AUC = 0.69) for DNA sequence-specific features, whereas RF and AdaBoost showed the best results (AUC = 0.81). Overall, the RF model, compared to other models, showed the best performance for different combinations of features and data sampling methods and was efficient on large datasets without overfitting. Therefore, the authors selected RF as the analysis model and predicted SEs using the top six features (Brd4, H3K27Ac, Cdk8, Cdk9, Med12 and p300) extracted by the integrated modeling of chromatin features. The results showed that Brd4 (AUC = 0.85) was slightly better than H3K27Ac for accuracy, with Cdk8 achieving an AUC of 0.84, which is comparable to that of H3K27Ac. Cdk9 showed an AUC of 0.83, Med12 showed an AUC of 0.83, and p300 showed an AUC of 0.76; however, after combining the top three features (Brd4, H3K27Ac and Cdk8), the model performed well with an AUC of 0.93. This implies that a combination of Brd4, H3K27Ac and Cdk8 is more effective in predicting SEs than each of them individually [81]. This method is called imPROSE (integrated methods for the prediction of SEs) and is available on the internet (Table 3).

**Table 3:** List of methods for SE identification using machine learning or methods for chromatin analysis containing SEs using machine learning

No.	Name of platforms	Description	Machine learning techniques	Datasets	URL	Reference
1	imPROSE	imPROSE is a computational workflow that predicts a SE or a component of an SE from a list of enhancer candidates. It also provides options for evaluating the predictive ability of individual enhancers and combinatorial factors. The pipeline integrates a variety of data including HM, DHS, TF, and DNA sequences. It has six parametric and non-parametric machine learning models: RF, SVM, k-NN, AdaBoost, DT, and NB.	RF, SVM, k-NN, AdaBoost, DT, NB	ChIP-seq [HMs (H3K27ac/ H3K4me1/ H3K4me3/ H3K9me3), RNA Pol II, CAs (p300/CBP), P-TFEb (Cdk9), Mediator (Med1/Med12/Cdk8), CRs (Brg1/Brd4/Chd7), Cohesin (Smc1/NipbL), Lsd1-NuRD (Lsd1/Mi2b), TFs (Oct4/Sox2/Nanog/Esrrb/Klf4/Tcfcp2l1/Prdm14/Nr5a2/Smad3/Stat3/Tcf3)], DNase-seq/DHS, RNA-seq, GFs [GC content/phastCons/repeat fraction]	http://asntech.github.io/improse/	[81]
2	MCIBox	MCIBox is based on a dimensionality reduction method and various clustering algorithms that can display multiple chromatin interactions at the single molecule level, allowing users to explore chromatin extrusion patterns and SE control modes in transcription, and identify single molecule clustered in microdomains’ chromatin complexes. MCIBox also incorporates a two-dimensional kernel density estimation algorithm that can automatically identify microdomain boundaries. MCIBox is an effective tool for the study of multiple chromatin interactions and has the potential to reveal previously unknown three-dimensional structures of the genome.	t-SNE, UMAP, PCA, ICA, Autoencoder, MDS, PHATE, *k*-means	ChIA-Drop, SPRITE, GAM	https://github.com/ZhengmzLab/MCIBox	[82]
3	DEEPSEN	DEEPSEN is a SE prediction method based on convolutional neural networks. This method integrates 36 different features and is capable of predicting genome-wide SEs. DEEPSEN can be used in conjunction with high-throughput experimental methods to improve the accuracy of SE predictions. The cases with three and four convolution layers (DEEPSEN-3L and DEEPSEN-4L) outperformed imPROSE in accuracy, repeatability, and the F1 score.	CNN	ChIP-seq [HMs (H3K27ac/ H3K4me1/ H3K4me3/ H3K9me3), RNA Pol II, CAs (p300/CBP), P-TFEb (Cdk9), Mediator (Med1/Med12/Cdk8), CRs (Brg1/Brd4/Chd7), Cohesin (Smc1/NipbL), Lsd1-NuRD (Lsd1/Mi2b), TFs (Oct4/Sox2/Nanog/Esrrb/Klf4/Smad3/Tcfcp2l1/Prdm14/Stat3/Tcf3/Nr5a2)], DNase-seq/DHS, GFs [AT content/GC content/phastCons/phastConsP/repeat fraction]	https://github.com/1991Troy/DEEPSEN	[53]
4	ResSEN	ResSEN is a novel solution for genome-scale SE prediction using supervised deep learning with residual neural networks. This model was created with the goal of improving the DEEPSEN method, optimizing CNN performance and avoiding the vanishing gradient problem. Even under conditions where overfitting was observed with the DEEPSEN method, no overfitting was observed with the ResSEN method, and the ResSEN method was superior to the DEEPSEN method in terms of accuracy.	Residual neural network	ChIP-seq [HMs (H3K27ac/ H3K4me1/ H3K4me3/ H3K9me3), RNA Pol II, CAs (p300/CBP), P-TFEb (Cdk9), Mediator (Med1/Med12/Cdk8), CRs (Brg1/Brd4/Chd7), Cohesin (Smc1/NipbL), Lsd1-NuRD (Lsd1/Mi2b), TFs (Oct4/Sox2/Nanog/Esrrb/Klf4/Smad3/Tcfcp2l1/Prdm14/Stat3/Tcf3/Nr5a2)], DNase-seq/DHS, GFs	N/A	[95]
5	DeepSE	DeepSE is a method based on a deep convolutional neural network model that aims to detect SEs from TEs. An important feature of DeepSE is that it can detect SEs using only DNA sequence information. The method also generalizes well to different cell lines.	CNN	DNA sequence	https://github.com/QiaoyingJi/DeepSE	[98]

*(continued)*

**Table 3:** Continued

No.	Name of platforms	Description	Machine learning techniques	Datasets	URL	Reference
6	HiC-Bench	The HiC-Bench method uses a 2D-fused lasso as a machine learning method to reproduce the Hi-C contact matrix, and then TAD boundaries can be classified based on their insulator scores. The results indicate that the higher the insulator score of the TAD boundary, the higher the CTCF level and that the score may vary among cell types. Furthermore, it was found that SEs are preferentially insulated by strong boundaries and that strong TAD boundaries and SE elements frequently co-occur in cancer patients.	2D-fused lasso	Hi-C, ChIP-seq [H3K27ac/CTCF], GFs [deletion/coduplication]	https://github.com/NYU-BFX/hic-bench	[99]
7	CHROMATIX	CHROMATIX is a computational model that deconvolves Hi-C data to reconstruct single-cell chromatin structures and identify important many-body interactions. This method allows for a large-scale quantification of the extent of third-, fourth-, and higher-order specific many-body interactions. The functional implications can also be elucidated by showing in detail how SEs, enhancers, promoters and other functional units stochastically assemble into spatial devices with measurable Euclidean distances.	Bayesian generative model	Hi-C	https://bitbucket.org/aperezrathke/chr-folder/src/master/	[109]

**Figure 3:**
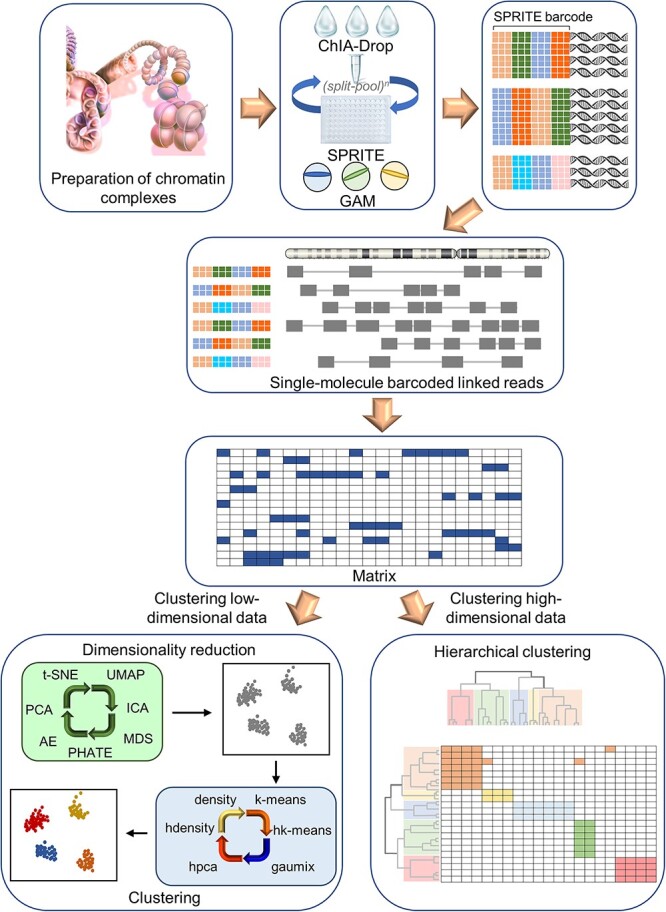
MCI-view workflow diagram based on Tian *et al.*’s study [82] MCI-view is a key component of MCIBox and provides a visualization system for multi-way chromatin interactions. Users can visualize their own experimental data by creating a formatted document containing complex information in each row from the results of multiple chromatin contacts generated by the multi-directional contact detection approach. MCI-view reads the data of the desired genomic region from the formatted input file, reconstructs the matrix, and either integrates a hierarchical clustering strategy to cluster the high-dimensional data directly or, if the features of the single molecule complex data are too complex, it performs a dimensionality reduction method to reduce the matrix data to low-dimensional data and further cluster them. This method was developed specifically as an integrated genomics viewer for multi-directional contact data. Abbreviations: GAM, genome architecture mapping; SPRITE, split-pool recognition of chromatin interactions by tag extension.

Tian *et al*. developed MCIBox, a toolkit for multiway chromatin interaction (MCI) analysis that includes visualization tools and a platform for identifying microdomains clustered in single-molecule chromatin complexes (Figure 3, Table 3) [82]. MCIBox is based on a dimensionality reduction method and various clustering algorithms that can display multiple chromatin interactions at the single molecule level, allowing users to explore chromatin extrusion patterns and SE control modes in transcription and identify microdomain clustered single-molecule chromatin complexes. MCIBox utilizes data obtained using three ligation-free technologies: ChIA-Drop, genome architecture mapping (GAM) and split-pool recognition of chromatin interactions by tag extension (SPRITE). ChIA-Drop is a microfluidic-based barcode-linked sequence for chromatin interaction analysis and is a simple method for analyzing multiple chromatin interactions with single molecule accuracy [83, 84]. By collecting three-dimensional proximity between any number of genomic loci, GAM eliminates many of the drawbacks associated with Chromosome Conformation Capture (3C)-based technologies [85, 86]. SPRITE is a genome-wide mapping technique for higher order interactions in the nucleus, allowing the genome-wide detection of interactions that occur at larger spatial distances [87, 88]. MCIBox’s approach to ChIA-Drop, GAM and SPRITE data is to utilize machine learning algorithms for dimensionality reduction or hierarchical clustering. As a result, a real-time exploration of chromatin topology structure and higher-order chromatin organization in gene transcription, chromatin extrusion patterns in chromatin organization and multiway chromatin interaction data is possible, but so is SE analysis. First, the ‘transcription regulation pattern view,’ a key component of MCIBox and a module of NCI-view for visualizing multiway chromatin interactions, can be applied to observe multidirectional chromatin interactions in SE regions. Hence, the configuration of SEs for gene regulation can be identified. MCI-view can also reveal SEs that interact at the single molecule level.

### Introduction of deep learning techniques to SE analysis

**Figure 4:**
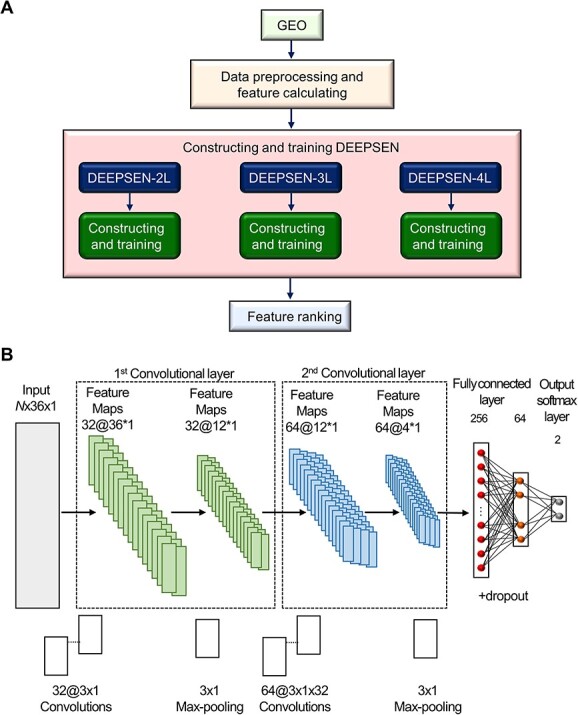
DEEPSEN workflow diagram and convolutional network architecture based on Bu *et al.*’s study [53]. (**A**) Gene Expression Omnibus (GEO) data were used, and data preprocessing and feature calculations were performed. Next, three models with different numbers of convolutional layers were constructed and trained. Pearson correlation coefficients were then evaluated to rank the features for predicting SEs. Finally, performance evaluation and analysis were conducted. (**B**) The architecture of DEEPSEN-2L is shown below, which consists of an input layer, a first convolution layer (including first max-Pooling), a second convolution layer (including second max-Pooling), a fully connected layer (including dropouts) and an output softmax layer. Convolution layer consists of two steps: convolution step and pooling step. The Rectified Linear Unit (ReLU) function was used as the activation function. Subsequent convolution layers capture the relationship between features extracted from subsequent layers to obtain higher-order features. Finally, the fully connected layer with dropouts transforms the input into a probability distribution by means of a softmax function. *N* indicates the number of samples.

Deep learning technology is being utilized in the medical field, particularly in medical image analysis [89–94], and for SE prediction, which we continue to introduce in this review. Bu *et al*. developed the algorithm DEEPSEN to predict SEs using convolutional neural networks (CNNs), a deep learning technique (Figure 4) [53]. The first step is data preprocessing and feature calculation, including DNA sequence composition features (AT content and GC content), histone modifications [H3K27Ac, H3K4me1, H3K4me3, and H3K9me3 (H3 lysine 9 tri-methylation)], transcription factors (Oct4, Sox2, Nanog, Esrrb, Klf4, Smad3, Tcfcp2l1, Prdm14, Stat3, Tcf3 and Nr5a2), RNA Pol II, DHS, coactivators (p300 and CBP), chromatin regulators (Brg1, Brd4 and Chd7), cohesin (Nipbl and Smc1), mediator complexes (Med1, Med12 and Cdk8), P-TFEb subunit (Cdk9) and the Lsd1-NuRD complex (Lsd1 and Mi2b). These are used to represent the SE using 36 different features. The second step is to build and train DEEPSEN; three models with different numbers of convolutional layers were built (two layers: DEEPSEN-2L, three layers: DEEPSEN-3L and four layers: DEEPSEN-4L). Subsequently, each model is trained using a backpropagation algorithm and a stochastic gradient descent optimization algorithm, and parameter tuning, and each model is validated using 5-fold cross validation [53]. The third step is feature ranking, which evaluates the contribution of each feature to the SE identification. To measure the predictive power of each feature, the Pearson correlation coefficient between each feature vector and the output label vector of all test samples is calculated to rank the individual features. Bu *et al*. reported that DEEPSEN-3L and DEEPSEN-4L outperform conventional methods in accuracy, repeatability and the F1-score [53]. Med12 (*r* = 0.746), Cdk8 (*r* = 0.731), Brd4 (*r* = 0.684), Cdk9 (*r* = 0.648), p300 (*r* = 0.618) and H3K27Ac (*r* = 0.605) showed particularly high correlations with the output label vectors of all test samples. The details of DEEPSEN are available on the web and can be used (Table 3).

Subsequently, aiming to improve the DEEPSEN method, Sabba *et al*. developed the ResSEN method using a residual neural network (ResNet) to optimize CNN performance and avoid the vanishing gradient problem (Table 3) [95]. ResNet is a method of learning deeper CNN by residual learning that introduces residual blocks to avoid degradation problems caused by vanishing gradient problems and other problems that occur when layers are deepened in CNNs [96, 97]. The use of skip connections on some layer blocks effectively simplifies the network and uses fewer layers in the initial learning phase. This reduces the effect of vanishing gradients and accelerates learning because fewer layers are propagated. In order to validate the performance of the ResSEN method, a comparison was made between the ResSEN and DEEPSEN methods using the same data as the DEEPSEN method, with 90% of the data for training and 10% of the data for testing. As a result, overfitting was observed in the DEEPSEN method but not in the ResSEN method [95]. The accuracy was also better when predicted by the ResSEN method, indicating that changing the algorithm used for ResNet increases the prediction accuracy of the SE [95].

**Figure 5:**
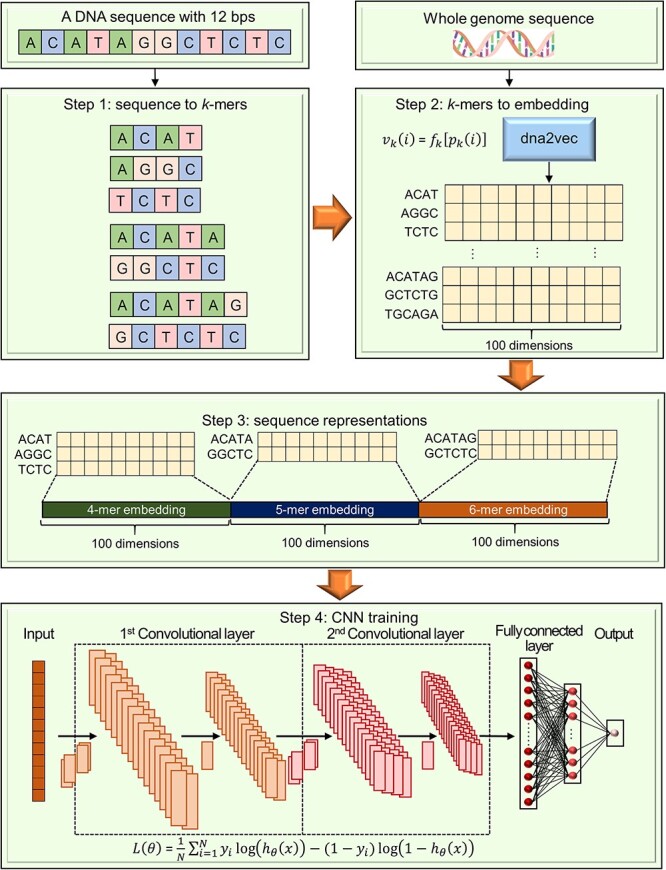
DeepSE workflow diagram based on Ji *et al.*’s study [98]. The *k*-mer sequence embedding was obtained by training dna2vec on the mouse and human genomes, respectively. Using this *k*-mer sequence embedding, the TE and SE sequences were represented by joining the sums of the corresponding *k*-mer embeddings. A CNN classifier was then trained on that array representation, and TE and SE were distinguished. Formula description: ${p}_k(i) (i=1,2,\dots, {4}^k)$ is the $i$th type of *k*-mer DNA sequence and a mapping from ${p}_k(i)$ to a fixed dimensional numeric vector was created using the dna2vec method. ${v}_k(i)$ is the fixed-dimensional representation of the $i$th type of *k*-mer DNA sequence and ${f}_k(.)$ a mapping from the *k*-mer DNA sequence to a fixed-length vector. The loss function in the CNN is a cross-entropy function, where ${y}_i$ is the label of the $i$th sample, ${h}_{\theta }(x)$ is the output of the neural network and *N* is the number of samples.

The detection of SEs is typically done using high-throughput data on various transcription factors and chromatin marks; however, we introduce a model that uses machine learning technology to detect SEs using only DNA sequences. Ji *et al*. [98] presented ‘DeepSE,’ a framework utilizing a deep CNN model that can detect SEs using only DNA sequence information (Figure 5). In this framework, the mouse and human genomes are first embedded into the *k*-mer sequence using dna2vec. Using this *k*-mer sequence embedding, TE and SE sequences are represented by joining the sum of the corresponding *k*-mer sequence embeddings. The sequence representation is then trained using a CNN classifier to classify TE and SE sequences. For predictive performance, six statistics are evaluated: accuracy ($\mathbf{ACC}=\left(\mathbf{True Positive (TP)}+\mathbf{True Negative (TN)}\right)/(\mathbf{TP}+\mathbf{TN}+\mathbf{False Positive (FP)}+\mathbf{False Negative (FN)})$), precision ($\mathbf{Pre}=\mathbf{TP}/\left(\mathbf{TP}+\mathbf{FP}\right)$), recall ($\mathbf{Rec}=\mathbf{TP}/\left(\mathbf{TP}+\mathbf{FN}\right)$), F1-score ($\mathbf{F}\mathbf{1}=\left(\mathbf{2}\mathbf{Pre}\times \mathbf{Rec}\right)/\left(\mathbf{Pre}+\mathbf{Rec}\right)$), the area under the receiver operating characteristic (AUROC) curve and the area under the precision-recall curve. DeepSE was trained on the mESC dataset and compared with the traditional method (imPROSE method), and DeepSE showed better performance on all six performance measures.

Enhancers clustered in the 12.5 kb range are integrated as stitched enhancers; however, if a stitched enhancer has a higher order signal, it is also considered an SE. Thus, using stitched enhancer datasets for five mouse cell types (mESCs, myotubes, macrophages, pro-B cells and Th cells), a classification between SEs and TEs was attempted using a model named DeepSE-specific, which is cell line-specific. The results showed that DeepSE-specific exhibited an AUROC of almost 0.9 in all five cell lines [98]. In addition, DeepSE-specific has been evaluated for three human cancer cell lines, H2171 (small cell lung cancer), U87 (glioblastoma) and MM1.S (multiple myeloma). The results showed that DeepSE-specific accuracy was 0.92 (H2171), 0.91 (U87) and 0.90 (MM1.S), while for AUROC, the performance was as high as 0.94 (H2171), 0.96 (U87) and 0.95 (MM1.S) [98]. These results suggest that DeepSE can generalize well among different cell lines and that cell type-specific SEs may share hidden sequence patterns among different cell lines. Since this method can detect SEs using only DNA sequence information, DeepSE is useful in its simple prediction manner. On the other hand, it only considers static information from the genome sequence. For example, it is difficult to detect developmental stage-specific SEs; therefore, existing methods that incorporate other types of features must be used. In this regard, we believe that the possibility of expanding the range of DeepSE applications and improving its accuracy should be evaluated in demonstration experiments, such as combining static genome sequence information with dynamic chromatin information (e.g. ChIP-seq). Indeed, the importance of establishing multimodal analysis methods using machine learning for the bioinformatics analysis of omics data has been highlighted in recent years [41, 42]. This approach is expected to produce a useful tool that can capture dynamic SE changes, such as developmental stages and disease outcome, by incorporating dynamic chromatin information into DeepSE methods and performing multimodal analysis. The details of DeepSE are available for use on the web (Table 3).

### Integrated analysis of SE and chromatin higher-order structure using machine learning

**Figure 6:**
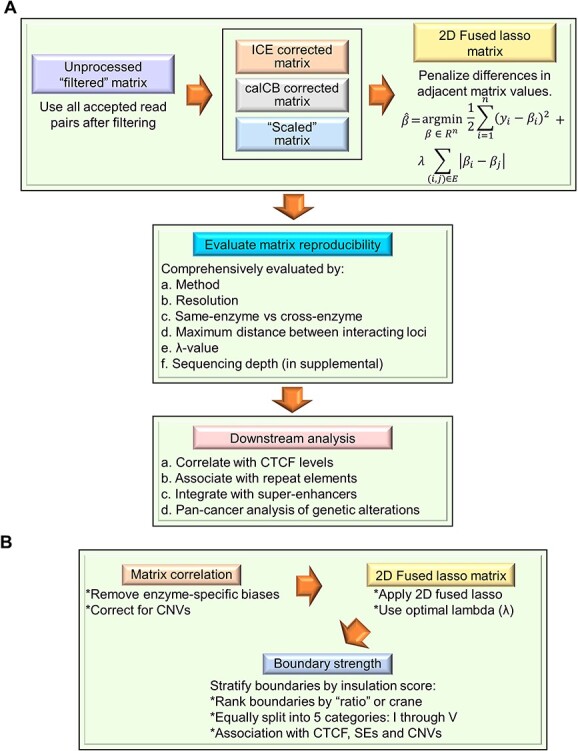
Workflow diagram for integrated analysis of SE and chromatin higher-order structures using machine learning based on Gong *et al.*’s study [99]. (**A**) This method uses untreated Hi-C contact matrices. First, processed Hi-C matrices are generated using iteratively corrected (ICE) ‘correction’, the ‘scaling’ approach developed by the authors. Then, calCB. 2D fused lasso is applied to the processed Hi-C matrices. Matrix reproducibility among biological replicates has been assessed across samples for various parameters using stratified adjusted correlation coefficients. Finally, downstream analyses include the characterization of TAD boundaries based on insulation strength, the enrichment of CTCF binding, proximity to repeat elements and SEs, and genetic alterations in cancer. (**B**) The workflow for stratifying TAD boundaries with insulation scores is shown. After processing the Hi-C matrices with ICE, calCB and scaling, 2D-fused lasso is applied at the optimal $\lambda$ value. Statistical significance is assessed using the Wilcoxon test among the distribution of stratified adjusted correlation coefficients among the chromosomes of a given sample for consecutive $\lambda$ values. Next, TAD calls and TAD boundary insulation score calculations are performed using the ‘ratio’ method developed by the authors, which classifies boundaries into five equal-sized categories. Formula description: 2D-fused lasso is used to achieve denoising by penalizing the difference between adjacent elements of the contact matrix via a penalty parameter $\lambda$. $y$ is the original (observed) contact matrix, and $\hat{\beta}$ is the optimized contact matrix such that the objective function is minimized. $E$ describes the adjacent elements of the matrix, $E$ = {$\left(i,j\right)$, where $i$ and $j$ are the adjacent elements of the matrix$\beta$}.

Machine learning has also been used for chromatin higher-order structure analysis, and Gong *et al*. used machine learning to determine the relationship between topological associating domain (TAD) boundaries and SE elements (Figure 6) [99]. TADs are chromosomal domains of approximately 1 Mb in length that are detected by Hi-C. Recently, it has become clear that transcription is regulated when enhancers located at distant sites within the TADs approach and act on target genes [100–102]. Although TADs are separated by conserved boundaries across cell types and species, the genome-wide properties of TAD boundary strength in mammals remain unclear. Therefore, Gong *et al*. [99] focused their research specifically on elucidating the function of the TAD boundary in cancer cells. The Hi-C method is an advanced version of the 3C method, which can detect any pair of genomic DNA fragments that are spatially close to each other in the three-dimensional structure of the genome in the cell nucleus using a next-generation sequencer and then estimate and analyze the three-dimensional genomic structure [103–105]. However, the reproducibility of the Hi-C contact matrix in the case of repeated experiments is unsatisfactory, and a calculation method is needed to improve this [99].

Hence, Gong *et al*. used two-dimensional (2D)-fused lasso, a form of machine learning, to remove noise by introducing a parameter *λ* that penalizes the difference between adjacent values in the Hi-C contact matrix. The results showed that the reproducibility of the Hi-C contact matrix was improved by appropriately adjusting *λ*, demonstrating the robustness of the noise reduction method using 2D-fused lasso [99]. Importantly, an integrated analysis of the strength of the TAD boundary with the SE confirmed that the SE is preferentially insulated by the strong boundary, suggesting that the SE and strong TAD boundary may be biologically related entities [99]. The frequency of structural changes involving strong TAD boundaries and SEs was investigated, and the overlap between strong TAD boundaries and SEs was significantly higher in cancer patients than between strong TAD boundaries and TEs [99]. This suggests that in cancer, the strong TAD boundary is not only protected from deletion but may also exist in duplicate with the SE. Furthermore, MYC, an oncogene overexpressed in cancer [106–108], was shown to be located next to a strong TAD boundary and to co-occur with several SEs in close proximity to the boundary [99]. Because machine learning technology excels at multimodal analysis and multitask learning, it is an important elemental technology in the development of such an integrated analysis platform.

Perez-Rathke *et al*. presented CHROMATIX, a computational model that deconvolves Hi-C data to reconstruct single-cell chromatin structures and identify important many-body interactions (Table 3) [109]. There is growing evidence that multivalent interactions play an important role in the formation of phase-separated, dense, functional chromatin assemblies in SEs, but the importance of spatial interactions in many-body (≥3) chromatin has not been assessed. CHROMATIX utilizes extensive biophysical folding simulations to estimate chromatin-multibody interactions from chromatin contact frequencies. By incorporating this inferred dependency into a Bayesian generative model, the method can decipher the intrinsic single-cell chromatin contact states underlying pairwise, population-averaged Hi-C data. This three-dimensional (3D) chromatin ensemble shows high consistency, with 96–97% Pearson correlation with measured Hi-C and spatial interaction frequencies across many loci [109]. The reconstructed 3D chromatin assemblies are generated from a very sparse set of interactions, and only ~5% of the predicted specific Hi-C interactions are able to generate polymer assemblies with contact frequencies consistent with Hi-C measurements [109]. The calculated 3D chromatin ensembles contain a wealth of structural information, allowing the prediction of highly non-random many-body (≥3) chromatin interactions. Importantly, the landscape of multisystem interactions derived from the analysis of 39 active genomic loci showed that SEs are enriched for participation in specific multisystem main loops compared to non-SE regions, and the overall level of SE-SE and SE-promoter interactions is high in certain multisystems [109]. These results can be judged as proof that, by using CHROMATIX, the computational SE interacts directly and nonrandomly spatially with other functional genomic regions in the many-body complex.

### Findings of our integrated analysis using imPROSE and DEEPSEN

Understanding the characteristics of the algorithm and performing appropriate data preprocessing and integration work with respect to the introduction of deep learning into the field of omics analysis such as genome and epigenome, (including SE analysis and the field of chromatin analysis) is crucial. We therefore postulated that comparison of the performance of each method when using the same dataset should be performed, given that the machine learning methods presented in this review article differ in terms of datasets and evaluation metrics. In this regard, we conducted a comparison of the accuracy between platforms using the same dataset and similar validation methods using imPROSE and DEEPSEN. The details of the data used in this comparative study are presented in the Supplementary Methods. In the original paper, k-fold cross validation was performed using similar datasets for both methods. However, since imPROSE shows 10-fold and DEEPSEN shows 5-fold cross validation results, we created five train/test datasets and evaluated the accuracy by 5-fold cross validation using the same train/test datasets in imPROSE and DEEPSEN (Figure S1 and Table S1). Our results demonstrated that, for AUC, NB in imPROSE was slightly more accurate, and DEEPSEN was comparable for all models (L2, L3 and L4). However, DEEPSEN was found to be more accurate than all imPROSE algorithms for the F1-score, which is the harmonic mean of Precision and Recall. In actual operation, DEEPSEN is considered more practical than imPROSE because SE is considered less frequent than TE. Importantly, we found problems related to imPROSE and DEEPSEN in this analysis, which are discussed in detail in the Discussion.

## DISCUSSION

In this review, we present the latest developments in the analysis of SEs using machine learning techniques and their application in medical biology. Because the analysis of SEs requires a genome-wide integration of multiple datasets [110–112], this is an area of research where the appropriate use of machine learning techniques can be effective. Particularly, as described in this review, several algorithmic analyses have been reported. However, SE is a new concept with a lack of cumulative findings to date; therefore, we strongly believe that continued research is needed in the future.

An important future aspect in SE analysis is the elucidation of molecular mechanisms in cancer cases in which no driver genes have been found. Although genomic medicine, which proposes optimal treatments based on genomic mutations, is currently attracting attention, especially in the field of oncology, only a limited number of patients actually benefit from genomic medicine [113–115]. There are a certain number of cancer patients for which no driver genes are found (also known as pan-negative types in lung cancer and melanoma) [116–121], which is a problem in promoting genomic medicine. This indicates the limitation of analyzing diseases by genomic variation alone with current technology. As described in this article, it has been suggested that oncogenes and SEs may co-occur and that oncogenes are activated in a SE-dependent manner [99]. Bearing this in mind, the identification of SEs is particularly important for cancer cases in which no driver genes have been found. Furthermore, genomic mutations in non-coding regions have also been reported to alter chromatin accessibility and affect gene expression in cancer cells [122–125]. Moreover, from the perspective of promoting precision medicine, it is necessary to verify the clinical usefulness of machine learning-based SE analysis in the future.

The current challenge regarding the analysis of SEs and the integration of analysis with other data is the need to perform multiple ChIP-seq and Hi-C analyses experiments; however, these analyses are complicated, require advanced techniques and have a high analysis cost [126–131]. Considering this, only a limited number of laboratories can realistically attempt to analyze a large number of clinical samples. The ‘DeepSE’ introduced in this article helps to solve this problem because it can detect SEs using only DNA sequences [98]. It remains important to develop highly accurate and versatile analysis methods by effectively implementing machine learning techniques such as DeepSE. In addition, for the analysis of SE, it is important to integrate the analysis with other omics’ data, such as whole genome analysis data and transcriptome analysis data, and with available information on diseases. Because machine learning technology excels at multimodal analysis and multitask learning, machine learning is considered an important elemental technology in the development of such an integrated analysis platform. However, while deep learning-based solutions have been published in many fields, especially in image analysis, and have outperformed conventional machine learning algorithms such as RF and SVMs, published results are still limited in the areas of omics analysis such as genome and epigenome, including SE analysis and chromatin analysis. The reason for this is that although the relationship between data in close proximity is important in the convolution and pooling stages of CNN, which is the core algorithm of deep learning, the relationship between data in close proximity is not necessarily high in the omics and chromatin analysis fields compared to images where this relationship is extremely high. For example, the TE/SE has a chromatin loop structure with genes, and even though the two-dimensional distance is far, they may be in close proximity in three dimensions. Altogether, we postulated that it is important to understand the characteristics of the algorithm and to perform appropriate data preprocessing and integration work with respect to the introduction of deep learning into the field of omics analysis such as genome and epigenome, including SE analysis and the field of chromatin analysis.

As the machine learning methods presented in this review article vary in terms of datasets and evaluation metrics, we conducted a comparison of the accuracy between platforms with the same dataset and similar validation methods, using imPROSE and DEEPSEN. We found that, for AUC, NB in imPROSE was slightly more accurate, and DEEPSEN was comparable for all models (L2, L3 and L4), but DEEPSEN was more accurate than all imPROSE algorithms for the F1-score. It is important to perform comparisons using the F1-score, rather than the AUC [132], because the data used are unbalanced (SE/TE = 0.0689). We found the following problems related to imPROSE and DEEPSEN in this analysis.

First, with respect to imPROSE, as mentioned above, the datasets used are unbalanced, so up-sampling by the synthetic minority over-sampling technique (SMOTE) [133] was used for imPROSE. In that case, the authors performed the SMOTE followed by train/test split [81]. This method is not recommended now because of the possibility of the leakage of information on the training data to the test data [134]. We have, therefore, attempted to correct this aspect of the analysis. As for DEEPSEN, in the original paper, the authors created a label sequence in which SE was converted to 1 and TE to 0 and a ulabel sequence in which 0 and 1 were exchanged in the label sequence (https://github.com/1991Troy/DEEPSEN/blob/54f5cbc59a621e27dc4628bbc65923c3deb0deec/4layer/improse-layer4_metrics.py#L37). The training was then performed with [label, ulabel] stored in that order, and the estimation results were output in a similar arrangement. The output results were then used to calculate the false-positive rate and true-positive rate for each threshold using the roc_curve function in the sklearn.metrics library (https://github.com/1991Troy/DEEPSEN/blob/54f5cbc59a621e27dc4628bbc65923c3deb0deec/output_4/auc.py#L20). In the code published by the authors, positive_label was not explicitly specified and the order of the output results was [SE probability, TE probability]; therefore, the accuracy evaluation for estimating TE was performed when it should have been for SE. We have consequently corrected this aspect of the analysis. This additional analysis confirms once again that comparing the accuracy of methods using the same data and similar validation methods is a useful tool when publishing a review paper as it often provides important and valuable insights. In addition, as a result of our own analysis using the two methods, we found new problems in both methods. Advancements in machine learning technology are rapid and have the potential to provide a continuous source of data-driven insights to optimize biomedical research, public health, and healthcare quality improvement. Furthermore, this is a newly and rapidly evolving academic field; therefore, in our humble opinion, we believe that it is important to continue to improve upon the published methods while suitably verifying them, as we did this time, to transform the healthcare industry for the better with its cutting-edge technology.

## CONCLUSIONS AND FUTURE PERSPECTIVES

In this review, we have introduced the latest findings on the analysis of SEs using machine learning techniques and their application to the field of medical biology and discussed current challenges and future directions. Whole genome analysis and other genome analyses are currently being conducted globally, particularly in the field of oncology, and their usefulness has been unequivocally established [135–144]. However, because the information obtained from genome analysis is limited, we believe that integrated analysis with SE analysis data and other omics data, such as transcriptome, is crucial in medical biology. Ideally, the development of platforms for the analysis of SEs and the integrated analysis of other data using machine learning should be vigorously pursued in this field, and we hope that this review will serve as a valuable contribution. Moreover, the analysis of SEs using machine learning techniques and their application to the field of medical biology is a newly emerging research area, and machine learning techniques are advancing rapidly. The fact that we found new problems with both of the previously published methods analyzed in this review paper is concerning. As machine learning promises a renaissance in the field of healthcare, it is necessary to proceed with caution. Consequently, we hope that this review, highlighting the possible concerns and future scope in integrated SE analytics, will be helpful to the researchers.

**Key Points**

- SEs strongly induce gene expression, and their abnormalities are involved in diseases, such as cancer.
- Machine learning techniques, including deep learning, have also been used to detect SEs, with results showing high accuracy.
- DeepSE, which uses the CNN, a type of deep learning, to detect SEs using only DNA sequences, has also been developed.
- The integrated analysis of SE data and chromatin structure data using machine learning suggested that, in cancer, SEs are sequestered by strong TAD boundaries and used as functional units within the cell, which may promote tumorigenesis.

## Supplementary Material

Supplementary_information_Hamamoto_et_al_021123_bbad107

## Notes

**Ryuji Hamamoto** is a Division Chief in the Division of Medical AI Research and Development, National Cancer Center Research Institute; a Professor in the Department of NCC Cancer Science, Graduate School of Medical and Dental Sciences, Tokyo Medical and Dental University and a Team Leader of the Cancer Translational Research Team, RIKEN Center for Advanced Intelligence Project. His research interests include medical AI, omics analysis and bioinformatics.

**Ken Takasawa** is a Postdoctoral Researcher in the Cancer Translational Research Team, RIKEN Center for Advanced Intelligence Project and an External Research Staff in the Medical AI Research and Development, National Cancer Center Research Institute. His research interests include medical applications of machine learning and bioinformatics.

**Norio Shinkai** is a PhD candidate in the Department of NCC Cancer Science, Graduate School of Medical and Dental Sciences, Tokyo Medical and Dental University. His research interests include bioinformatics, omics analysis and machine learning applications in medicine.

**Hidenori Machino** is a Postdoctoral Researcher in the Cancer Translational Research Team, RIKEN Center for Advanced Intelligence Project and an External Research Staff in the Medical AI Research and Development, National Cancer Center Research Institute. His research interests include gynecologic oncology and bioinformatics.

**Nobuji Kouno** is a PhD candidate in the Department of Surgery, Graduate School of Medicine, Kyoto University. His research interests include gastroenterological surgery, medical AI and bioinformatics.

**Ken Asada** is a Research Scientist in the Cancer Translational Research Team, RIKEN Center for Advanced Intelligence Project and an External Research Staff of Medical AI Research and Development, National Cancer Center Research Institute. His research interests include omics analysis, bioinformatics and medical AI.

**Masaaki Komatsu** is a Deputy Team Leader in the Cancer Translational Research Team, RIKEN Center for Advanced Intelligence Project and an External Research Staff of Medical AI Research and Development, National Cancer Center Research Institute. His research interests include obstetrics and gynecology, medical AI and bioinformatics.

**Syuzo Kaneko** is a Laboratory Head in the Division of Medical AI Research and Development, National Cancer Center Research Institute and a Visiting Scientist in the Cancer Translational Research Team, RIKEN Center for Advanced Intelligence Project. His research interests include epigenomics, bioinformatics and medical AI.
