# Supplementary material for: Analysis of super-enhancer using machine learning and its application to medical biology
Source: Brief Bioinform. 2023 Mar 23;24(3):bbad107. doi: 10.1093/bib/bbad107 (PMC10199775; doi:10.1093/bib/bbad107)
Supplement: Supplementary_information_Hamamoto_et_al_021123_bbad107 [file supplementary_information_hamamoto_et_al_021123_bbad107.zip › Supplementary_information_Hamamoto_et_al_021123_bbad107.docx]

**Supplementary information**

**Analysis of super-enhancer using machine learning and its application to medical biology**

The file contains

Supplementary Methods

Supplementary Figures S1

Supplementary Table S1

Reference

**Supplementary Methods**

**Integrated analysis using imPROSE and DEEPSEN**

We used the pre-integrated dataset (https://github.com/asntech/improse/blob/master/improse/data/original_data.csv) provided in the imPROSE GitHub repository. The developers of the imPROSE, Khan, A. and Zhang, X., created and provided an integrated dataset of 32 mouse ESCs obtained from the Gene Expression Omnibus and genomic features obtained from the UCSC table browser [1]. The integrated dataset consisted of the following data: histone modification data (H3K27ac, H3K4me1, H3K4me3, and H3K9me3), DNaseI, RNA Pol II, transcriptional co-activating proteins (p300 and CBP), P-TFEb subunit (Cdk9), sub-units of the Mediator complex (Med1, Med12, and Cdk8), other chromatin regulators (Brg1, Brd4, and Chd7), Cohesin (Nipbl and Smc1), subunits of Lsd1-NuRD complex ( Hdac1, Hdac2, Lsd1, and Mi2b), 11 TFs (Oct4, Sox2, Nanog, Smad3, Stat3, Tcf3, Esrrb, Klf4, Prdm14, Tcfcp2I1, and Nr5a2), and genomic features (AT_content, GC_content, phastCons content, phastCons, phastConsP, repeat_fraction, and length).


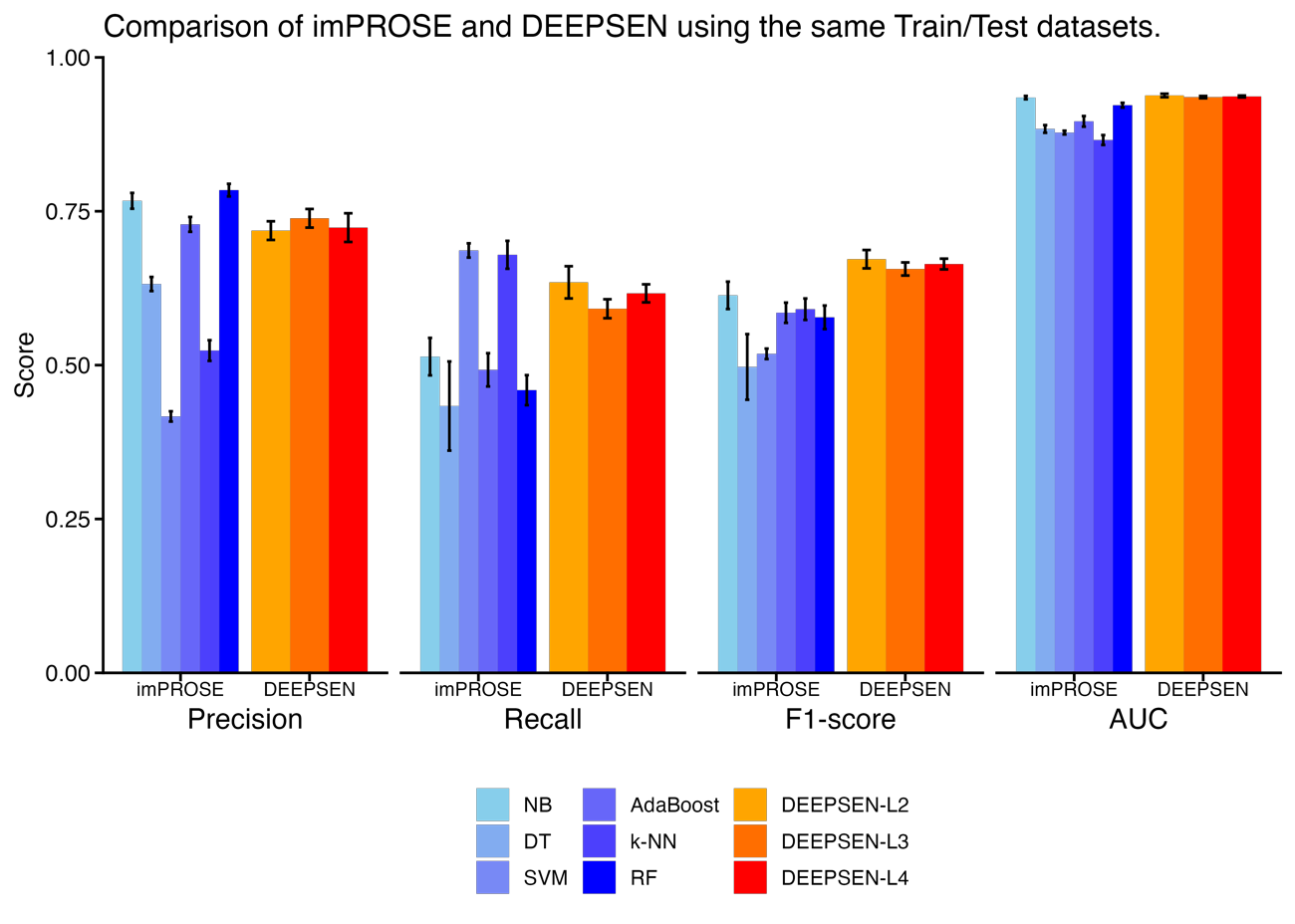
**Supplementary Figure S1.** Bar graphs comparing the metrics of 5-fold cross validation for imPROSE and DEEPSEN, respectively. The 5-fold cross validation was performed with a fixed random state, and the same Train /Test datasets are used for both methods. Blue bars indicate each machine learning model in imPROSE, red bars indicate models with different number of convolutional layers in DEEPSEN. Error bars indicate standard errors.

**Supplementary Table S1. List of the metrics of the imPROSE and the DEEPSEN.**

|  | Model | Metrics | | | |
| --- | --- | --- | --- | --- | --- |
|  |  | Precision | Recall | F1-score | AUC |
| imPROSE | AdaBoost | 0.727 ± 0.076 | 0.480 ± 0.046 | 0.577 ± 0.042 | 0.887 ± 0.023 |
|  | DT | 0.652 ± 0.024 | 0.444 ± 0.044 | 0.528 ± 0.033 | 0.885 ± 0.013 |
|  | k-NN | 0.522 ± 0.026 | 0.657 ± 0.023 | 0.581 ± 0.014 | 0.860 ± 0.015 |
|  | SVM | 0.761 ± 0.024 | 0.516 ± 0.046 | 0.614 ± 0.036 | 0.931 ± 0.013 |
|  | NB | 0.417 ± 0.020 | **0.696 ± 0.028** | 0.521 ± 0.022 | 0.878 ± 0.010 |
|  | RF | **0.799 ± 0.043** | 0.462 ± 0.037 | 0.584 ± 0.027 | 0.920 ± 0.017 |
| DEEPSEN | DEEPSEN-L2 | 0.727 ± 0.043 | 0.596 ± 0.032 | 0.654 ± 0.022 | 0.936 ± 0.010 |
|  | DEEPSEN-L3 | 0.716 ± 0.045 | 0.610 ± 0.030 | 0.658 ± 0.028 | 0.935 ± 0.011 |
|  | DEEPSEN-L4 | 0.705 ± 0.050 | 0.635 ± 0.033 | **0.667 ± 0.022** | **0.938 ± 0.011** |

*The values show the mean and standard error of 5-fold cross validation, and the bold font indicate the best score of each metric.

**Reference**

1. Rosenbloom KR, Armstrong J, Barber GP et al. The UCSC genome browser database: 2015 update. *Nucleic acids research* 2015;**43**(D1):D670-D81.
